# Diagnosis of Thymic Clear Cell Carcinoma by Cytology

**DOI:** 10.1155/2013/617810

**Published:** 2013-09-23

**Authors:** Seema A. Lale, Patricia G. Tiscornia-Wasserman, Mohamed Aziz

**Affiliations:** ^1^Mercy Hospital, 800 Myrtle Street, Independence, KS 67301, USA; ^2^Cytopathology, Columbia University Medical Center, 630 West 168th Street, PH15-1586, New York, NY 10032, USA; ^3^Cytopathology, Hofstra North Shore-LIJ School of Medicine and at New York Medical College, 6 Ohio drive, Lake Success, NY 11042, USA

## Abstract

Clear cell carcinoma of the thymus is a rare tumor. Few cases of clear-cell carcinoma of thymus have been documented (Truong et al., 1990 and Wolfe III et al., 1983). All these cases were diagnosed by histopathological examination of the tissue. Diagnosis of thymic clear cell carcinoma on cytology is extremely challenging. Here we report the first case of thymic clear cell carcinoma diagnosed by cytological examination of the pericardial fluid with the help of immunocytochemistry. Differential diagnosis included adenocarcinoma, mesothelioma, and thymic clear cell carcinoma. Thymic carcinoma with clear cell features has an aggressive clinical behavior including our case, where it was already metastasized at the time of presentation.

## 1. Introduction

Tumors of the thymus include thymoma, thymolipoma, carcinoid tumors, germ cell tumors, malignant lymphoma, and secondary involvement of the thymus by carcinoma [1]. Thymic carcinoma is a thymic epithelial tumor with a high degree of histologic anaplasia, obvious cell atypia, and increased proliferative activity and is unassociated with immature T cells [2, 3]. Because of the relative rarity of thymic carcinoma, wide variation of histology, and the differences in diagnostic criteria, its exact incidence in any given country and geographic differences in frequency are unknown. The largest series is made up of all consultation cases [4]. The largest series from a single institute is comprised of 20 cases collected over 75 years. 

The low-grade malignancy group of thymic carcinoma includes well-differentiated squamous cell carcinoma, mucoepidermoid carcinoma, and basaloid carcinoma. The high-grade malignancy group includes lymphoepithelioma-like carcinoma, small cell/neuroendocrine carcinoma, clear cell carcinoma, sarcomatoid carcinoma, and undifferentiated/anaplastic carcinoma. We describe a case of thymic clear cell carcinoma diagnosed by cytology. To our knowledge, this is the first case of clear cell carcinoma of the thymus diagnosed by cytology with the help of cytology and immunocytochemistry.

## 2. Case Report

A 66-years-old female was referred to our hospital by her cardiologist to manage a bloody pericardial effusion. The patient reported to her cardiologist with chest pain and difficulty in breathing. The patient was diagnosed with cardiac tamponade. Pericardial tap was performed, and it produced 90 mL of thick bloody fluid. The patient had a surgical history of previous cholecystectomy and tonsillectomy. No other significant medical history was known at the time of presentation. Cytology smears and cell-block preparation were prepared.

## 3. Materials and Methods

The fluid was transported to the cytology laboratory for immediate cytospin processing followed by the application of both Diff-Quik (Medical Chemical Corp., Torrance, CA, USA) and Papanicolaou stains (Cardinal Health, Ontario, Canada). The residual pellet, after cytospin processing, was fixed in 10% buffered neutral formalin for cell block preparation followed by hematoxylin and eosin (H&E) staining and ancillary studies. 

Immunocytochemistry stains were performed on unstained sections of formalin fixed, paraffin embedded cell block by the standard avidin-biotin technique. The panel of antibodies used included cytokeratin 7 (CK7) cytokeratin 20 (CK20), cytokeratin 5/6 (CK5/6) epithelial membrane antigen (EMA), TTF-1, Calretinin, B72.3, BER EP4, CD 99, CD 117, P63, E-Cadherin, ER, CA 125, and CA 19.9. Special stains included mucicarmine and PAS with diastase. 

## 4. Results

**Figure 1:**
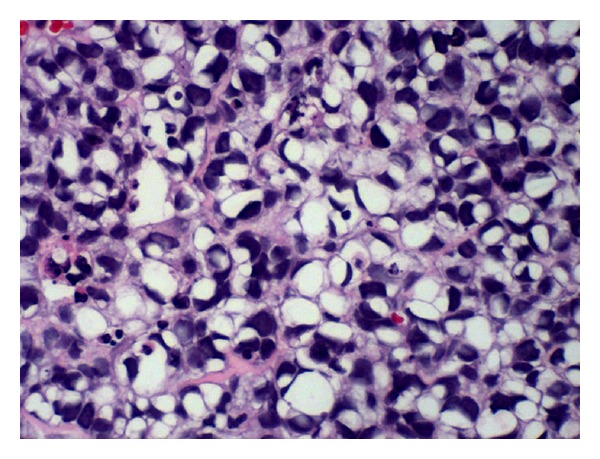
H & E stained cell block (40x).

**Figure 2:**
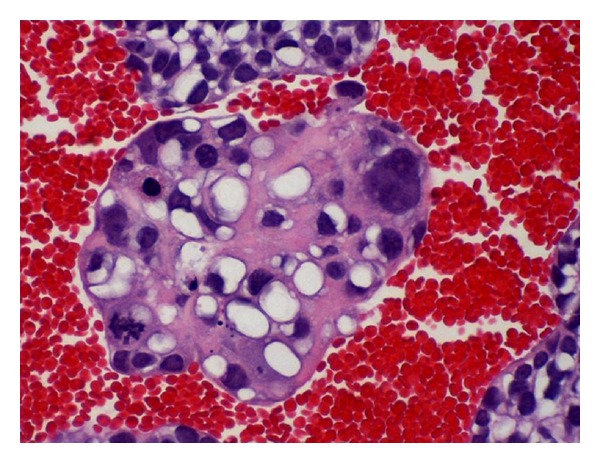
H & E stained cell block (40x).

**Figure 3:**
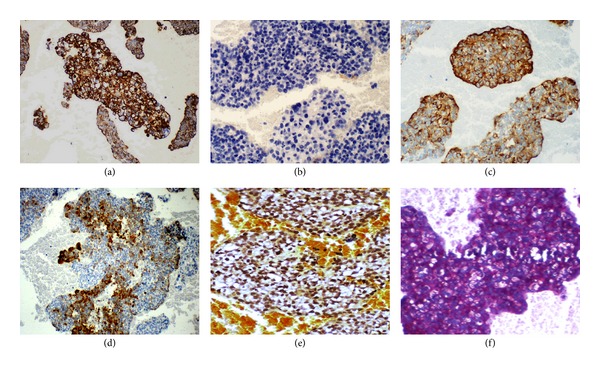
Ancillary studies of CK7, CK20, EMA, CK5/6, Mucin, and PAS.

The cell block preparation (Figures 1 and 2) showed a cellular tumor composed of clusters of highly malignant cells with focal glandular arrangement. Many cells revealed vacuolated cytoplasm, suggestive of signet ring appearance. Tumor cells revealed mitosis of more than ten per ten high power field. Immunocytochemical stains confirmed the epithelial origin of the tumor (CK7, EMA positive, and CK20 negative). Mucicarmine stain was negative, while PAS with diastase was positive in nonvacuolated cells, indicating the presence of glycogen, but not mucin (Figures 3(a)–3(f)). Tumor cells were focally positive for CA 19.9, CA 125, E-Cadherin, BER EP4, and CK5/6. Immunostains for TTF-1, Calretinin, B72.3, ER, P63, CDX-2, CD 99, and CD 117 were negative. This immunoprofile was most consistent with the rare variant of thymic carcinoma, the clear cell type. 

## 5. Discussion

Thymic carcinoma is a relatively rare tumor. The largest series from a single institution comprises of 20 cases collected over 75 years at the Mayo clinic [2]. The 13 cases reported by Truong et al. were collected from 2 hospitals over a 22-year period [1].

Clear cell carcinoma is a rare subtype of thymic carcinoma. It was first reported by Snover et al. in 1982 [5]. According to Hasserjian et al., the clear cell phenotype represents a secondary change superimposed on typical thymic cancer [6]. Histology of the tumor reveals lobules or sheets of polygonal cells with clear cytoplasm, small nuclei with inconspicuous nucleoli, and a scanty fibrovascular stroma. The clear cytoplasm contains abundant glycogen but no mucin. Electron microscopy studies suggested that this tumor is a variant of squamous cell carcinoma due to the presence of tonofibrils, desmosomes, and high molecular weight keratins [1]. It may also occur in combination with type A thymoma, squamous cell carcinoma, or undifferentiated carcinoma of the thymus. 

Thymic clear cell carcinoma is difficult to diagnose because of ill-defined symptoms and is often diagnosed incidentally [6]. Other mediastinal tumors like seminomas also present clinically in the same way [5, 7]. It has aggressive clinical behavior, and metastases are frequent. The most frequent sites of metastases are lymph nodes (mediastinal, cervical, and axillary), followed by bone, lung, liver, and brain. Factors associated with the prognosis are the histologic type of carcinoma, circumscription of the tumor, lobulation, and mitotic activity [4]. Our patient presented with bloody pericardial effusion without any known history at the time of presentation. Clear cell features have been described in a variety of organs, most commonly in the kidney, ovary, lung and also in the pancreas and thyroid [8, 9]. Metastasis from renal clear cell carcinoma or ovarian clear cell carcinoma should be excluded in patients with thymic clear cell carcinoma. Differential diagnosis of the metastatic tumor in pericardial fluid was metastatic adenocarcinoma including lung, metastatic malignant mesothelioma, and thymic carcinoma. Immunocytochemical stains, mucicarmine, and PAS with diastase ruled out mesothelioma and adenocarcinoma and supported the thymic cell carcinoma with clear cell features. The patient was then clinically investigated, and the CT scan of the chest revealed a large heterogeneous right anterior mediastinal mass with punctate calcifications, measuring 4.7 × 3.7 × 3.0 cm, with pericardial extension.

Thymic clear cell carcinoma is considered to be a tumor of high grade histology in spite of its relatively slow growth. 13 cases of thymic carcinoma with clear cell features have been reported. Eight occurred in men and 5 in women [6, 10–12]. The mean age at diagnosis was 53.5 years (range 33 to 69) in men and 53.4 years (range 36 to 84) in women with the youngest patient presenting at 33 years of age. In 7 patients the clinical history was available and presented with nonspecific symptoms of chest pain or dyspnoea. Our patient also presented with medical cardiac symptoms. Histology on all of these cases revealed cytoplasmic clearing, mainly due to glycogen accumulation. Periodic acid Schiff (PAS) stain was performed in 12 out of thirteen cases and was positive in nine cases as in our case. All these 13 cases were diagnosed histologically either by biopsy or surgical resection.

Clinical a followup showed that 5 out of these 13 patients developed metastasis. Our patient presented with metastatic pericardial effusion. This reveals the aggressive clinical behaviour of thymic carcinoma with clear cell features. 

A review of literature the of thymic clear cell carcinomas revealed that 9 of 13 patients either died of the disease or had persistent disease at the time of followup. 

 Treatment for thymic cancer remains to be established. Complete resection is the best way to a cure but is often impossible, as in our case due to pericardial extension. Preoperative chemotherapy has been recommended to reduce the volume of a tumor to allow complete resection and to increase the survival rate [13]. 

Our patient was treated systemically with 4 cycles of cytoxan, adriamycin, and carboplatin. The tumor was then resected, and histology confirmed the diagnosis of thymic carcinoma with clear cell features. 

Prognostic factors in general include race, sex, age, immunologic status, therapeutic effects, stage, and tumor histology. At the histologic level, the most important prognostic factor is the histologic type of thymic carcinoma. Clear cell carcinoma may also be considered as a form of poorly differentiated squamous cell carcinoma. The cytologic atypia and mitotic index are related to the degree of histological differentiation [14]. 

Diagnosis of thymic clear cell carcinoma on cytology is extremely difficult. The fine needle aspiration biopsy specimen is characterized by clearly malignant appearing tumor cells. Except for the lymphoepithelioma-like pattern, thymic carcinomas usually lack the dual cell population characteristic of other types of thymomas. Pronounced cytological atypia, prominent nucleoli, and numerous or bizarre mitotic figures are important diagnostic features of thymic carcinoma. Necrosis, blood, and scattered inflammatory cells may be seen in the background of the smear. Tumor cells in thymic clear cell carcinoma are usually uniform in appearance and have abundant, clear cytoplasm. Though the cellular pleomorphism is present to warrant the diagnosis of carcinoma, nuclear atypia is not markedly present. Nuclear chromatin is finely dispersed and nucleoli are usually not prominent. Mucicarmine stain is characteristically negative. PAS positive, diastase sensitive material consistent with glycogen, is present in the cytoplasm of tumor cells. Tumors are uniformly reactive for low and high molecular weight keratins and EMA.

 The generally poor prognosis is influenced by the diagnostic difficulty with ill-defined symptoms leading to large occult tumor growth with involvement of vital structures at the time of presentation. 

Thymic clear cell carcinoma is a challenging cytologic diagnosis, making it more difficult when it has already been metastasized without the history of any primary tumor. We would like to emphasize the importance of special stains like mucicarmine and PAS with diastase along with immunocytochemistry on cytology specimens to make a correct diagnosis. This was the first case of thymic clear cell carcinoma diagnosed on cytology with the help of special stains and immunocytochemistry.
